# Mental Health of Medical Students in Portugal: The Role of Sexual Orientation

**DOI:** 10.3390/healthcare14132023

**Published:** 2026-07-07

**Authors:** Ana Moura, Mateus Melo Ferreira, Vânia D’Alva-Teixeira, Rui Macedo, Pedro Morgado

**Affiliations:** 1 Life and Health Sciences Research Institute (ICVS), School of Medicine, University of Minho, Campus de Gualtar, 4710-057 Braga, Portugal; a96407@alunos.uminho.pt (A.M.); a88888@alunos.uminho.pt (M.M.F.); vaniadalvateixeira@gmail.com (V.D.-T.); rui.macedo@ulsb.min-saude.pt (R.M.); 2 ICVS/3B’s—PT Government Associate Laboratory, 4710-057 Braga, Portugal; 3 2CA-Braga—Clinical Academic Center of Braga, Hospital de Braga, 4710-243 Braga, Portugal

**Keywords:** anxiety, burnout, depression, distress, LGBTQ+, medical students

## Abstract

**Highlights:**

**What are the main findings?**

- LGBTQ+ medical students report more difficulties in personal, academic, social, and financial areas and show higher levels of distress than heterosexual students.
- Burnout, low-grade satisfaction, and substance abuse correlated with anxiety and depression levels differently depending on sexual orientation.

**What are the implications of the main findings?**

- Higher education institutions should develop and implement policies to reduce distress among students, especially those with greater vulnerabilities and less social support, such as LGBTQ+ individuals.
- Both universities and medical schools to actively create policies promoting inclusion, visibility, and normalization of diversity to foster a more welcoming academic environment.

**Abstract:**

Background/Objectives: Depression and burnout are common among medical students, leading to serious academic and professional consequences. LGBTQ+ students are especially vulnerable to mental health issues. This study aims to help understand the associations between sexual orientation and distress levels among Portuguese medical students. Methods: An observational, analytical, cross-sectional study was conducted with participants from Portuguese medical schools. Data collection took place between 2022 and 2025 through electronic questionnaires. Assessment tools included the Multidimensional Scale of Perceived Social Support, the Beck Depression Inventory, the State-Trait Anxiety Inventory, and the Maslach Burnout Inventory Student Survey. Statistical significance was set at *p* < 0.05 (95% confidence interval). Group comparisons by sexual orientation were conducted using chi-square/Fisher’s exact tests, Mann–Whitney U tests, and independent samples t-tests. Predictors of depression and state anxiety were assessed using multiple linear regression. Results: The sample included 1668 (83.2%) heterosexual students and 336 (16.8%) LGBTQ+ students. The rates of depression and anxiety were 43.2% and 25.7%, respectively. LGBTQ+ students reported more difficulties in personal, academic, social, and financial areas and showed higher levels of distress than heterosexual students. Key factors linked to distress included burnout, social support, and grade satisfaction. Additionally, burnout, low-grade satisfaction, and substance abuse were associated with anxiety and depression levels differently depending on sexual orientation. Conclusions: This study’s findings confirm higher distress levels among the LGBTQ+ group compared to the heterosexual group, helping us identify students who are more vulnerable to developing mental illness and prompting us to reconsider which aspects of medical training and culture contribute to this distress.

## 1. Introduction

Psychological distress, often referred to as distress, is a state of suffering that involves symptoms of anxiety, depression, and burnout [1]. The prevalence of distress among medical students is high, and this group is at increased risk of developing mental illness [2,3]. Persistent distress can lead to serious consequences, such as lower academic performance, increased risk of dropping out, and, in severe cases, suicidal thoughts [3,4]. Furthermore, if these symptoms are not treated promptly, they can persist during clinical practice and impact the quality of healthcare provided [4]. Clinicians in distress display less empathy and professionalism [5,6]. Therefore, early identification of distress and its triggering factors becomes crucial [7].

The causes of distress in medical students are multifactorial, including personal, social, economic, and academic factors, so the risk can begin even before the start of the course [8,9]. Sexual minorities are consistently described as being at increased risk of mental health problems, largely due to minority stress processes, including experiences of stigma, discrimination, prejudice, and internalized negative attitudes [10]. Within this context, LGBTQ+ medical students may represent a particularly vulnerable subgroup. In addition to the academic demands and psychological burden inherent to medical training, LGBTQ+ students may face identity concealment, fear of discrimination, and reduced social support, factors that have been associated with higher levels of depression, anxiety, burnout, and psychological distress compared with their heterosexual peers [1,9,11]. International studies show that LGBTQ+ medical students experience higher levels of distress compared to heterosexual medical students [11,12,13,14,15,16,17]. In Portugal, non-heterosexual orientation has already been identified as a potential risk factor for worse levels of distress [9]. However, studies in this area are scarce and do not allow us to understand the relationship between LGBTQ+ sexual orientation and the factors that make this population vulnerable in the Portuguese context.

Therefore, this study aims to analyze distress among medical students in Portugal, comparing students of different sexual orientations across sociodemographic, academic, financial, social, and distress factors. All of these variables are going to be explored because these factors are widely recognized in the literature as critical structural determinants of student well-being [9]. The present study also seeks to identify the factors that contribute to higher levels of distress in this group, helping to understand what makes LGBTQ+ medical students more vulnerable. The data collected will be very valuable for choosing intervention strategies and developing an early detection plan for medical schools.

## 2. Materials and Methods

### 2.1. Type of Research Study

A cross-sectional analytical observational study was conducted at the University of Minho School of Medicine, a public medical school in Portugal.

A cross-sectional analytical observational design was selected as it provided an efficient baseline to determine the prevalence of psychological distress and examine associations between contextual factors and mental health outcomes simultaneously. Furthermore, the single-point, completely anonymous nature of the survey protected participant privacy regarding a sensitive topic (sexual orientation) and encouraged participation.

### 2.2. Population and Sample

All students enrolled in an Integrated Master’s in Medicine during the 2022/2023, 2023/2024, and 2024/2025 academic years at one of the nine Portuguese medical schools offering a full cycle of studies were invited to participate. The invitation was disseminated through institutional academic channels, and participation was voluntary and anonymous. Therefore, the final sample corresponds to a non-probability convenience sample based on voluntary response. The schools included are as follows:

- School of Medicine of the University of Minho, Braga;
- Faculty of Medical Sciences of the New University of Lisbon, Lisboa;
- Faculty of Health Sciences of the University of Beira Interior, Covilhã;
- Faculty of Medicine of the Católica Medical School, Sintra;
- Faculty of Medicine and Biomedical Sciences of the University of Algarve, Faro;
- Faculty of Medicine of the University of Lisbon, Lisboa;
- Faculty of Medicine of the University of Coimbra, Coimbra;
- Faculty of Medicine of the University of Porto, Porto;
- Abel Salazar Institute of Biomedical Sciences of the University of Porto, Porto.

At the University of Algarve’s Faculty of Medicine and Biomedical Sciences, the Integrated Master’s in Medicine lasts four years, with the first through fourth years corresponding to the third through sixth years at other medical schools. To simplify written language, these students were statistically grouped into those latter categories. Católica Medical School agreed to participate in the study in 2023 and 2024.

### 2.3. Data Collection Process

The questionnaires were administered electronically using Google Forms and distributed via institutional email, academic year groups, and student communication channels at each Medical School. Data collection took place during the 2022/2023, 2023/2024, and 2024/2025 academic years. The survey link remained open for a defined collection period in each academic year, with at least one reminder sent during the data collection window to increase participation. Participation was anonymous, voluntary, and no incentives were offered.

Data collection was conducted by V.D.T., M.M.F., and A.M., corresponding to each academic year. Since the purpose of this study was to perform a cross-sectional analysis, 157 responses with repeated participant codes across years were excluded, retaining only each student’s most recent participation.

### 2.4. Ethical Considerations and Confidentiality

The original study and the addendum, which authorized national data collection, were approved by the Ethics Subcommittee for Life and Health Sciences at the University of Minho (CEICVS 064/2015). Data confidentiality was maintained throughout the study. To facilitate potential longitudinal follow-up of the sample, a participant code was created using the following key: number of siblings + the first letter of the mother’s first name + the first letter of the father’s first name + the month of the mother’s birth.

### 2.5. Assessment Instruments

The questionnaire covers questions about personal, economic, family, academic, and social factors. Personal and Sociodemographic Variables include Sex, Age, and Sexual Orientation. Sexual orientation was assessed via a single-choice question asking participants to identify as Heterosexual, Bisexual, Homosexual, or Other. For statistical viability in comparative analyses, these were further categorized into Heterosexual and LGBTQ+ groups.

Regarding Socioeconomic and Family Background, parental education was measured separately for mothers and fathers using the standard Portuguese academic cycles (from primary education to higher education degrees). Parental employment status was collected as a categorical variable (e.g., Employed, Unemployed, Retired). Financial aid status was determined by a binary question regarding whether the student held a government scholarship (Yes/No).

Academic and Living Context: Curricular year, medical school affiliation, and university entry preference order (e.g., 1st option, 2nd option) were recorded. Living arrangements were assessed by asking if the student had relocated to attend university and with whom they currently cohabited (e.g., Living with family, Living alone/with peers).

Psychosocial Stressors and Substance Use: Past or current utilization of psychological or psychiatric support was assessed as a binary outcome (Yes/No). Recent negative life events were recorded using an itemized checklist of common acute stressors. Finally, perceived daily difficulties across 13 distinct life domains (including academic performance, financial strain, and relationships) and the frequency of substance use (alcohol, tobacco, and other drugs) were also measured.

The questionnaire also includes the following assessment tools.

#### 2.5.1. Multidimensional Scale of Perceived Social Support (MSPSS)

The MSPSS, developed by Gregory Zimet and colleagues, was created to assess social support from family, friends, and significant others [18]. The scale was validated for the Portuguese population by Serafim Carvalho and collaborators [19]. MSPSS includes 12 items: four assess perceived social support from family, four from friends, and four from significant others, providing an overall measure of total social support. Each subscale score is calculated as the mean of its four items, while the total score is derived from the mean of all 12 items [19]. The maximum score for each subscale, as well as for the total scale, is 7 [18].

#### 2.5.2. Beck’s Depression Inventory (BDI)

The BDI is a 21-item scale developed by Aaron T. Beck and colleagues, adapted for the Portuguese population by Adriano Vaz Serra and collaborators [20,21]. The scale evaluates affective, cognitive, motivational, and physical domains, measuring depressive symptom severity from 0 to 63 [22]. The established cut-off points are no depressive symptoms (up to 12 points), mild depression (13 to 18 points), moderate depression (19 to 24 points), and severe depression (25 points or more) [23].

#### 2.5.3. State-Trait Anxiety Inventory (STAI)

The STAI-Y inventory, developed by Charles Spielberger and translated into Portuguese by Danilo Silva, includes two 20-item self-report scales: STAI-Y1, which measures transient state anxiety, and STAI-Y2, which assesses stable trait anxiety as a personality trait [22,24,25]. The scores range from 20 to 80, with higher scores indicating greater levels of either state or trait anxiety. The 75th percentile was used as the cutoff point (scores ≥ 57 on the STAI-Y1 and STAI-Y2, respectively) to differentiate cases with increased symptomatology from those without [7]. There are 10 reverse-scored items in the STAI-Y1 (questions 1, 2, 5, 8, 10, 11, 15, 16, 19, 20) and 9 in the STAI-Y2 (questions 21, 23, 26, 27, 30, 33, 34, 36, 39). The STAI-Y1 scale was administered first to prevent any interference with the responses provided [25].

#### 2.5.4. Maslach Burnout Inventory—Students Survey (MBI-SS)

The MBI-SS is an inventory adapted for use with students by Shaufeli and for the Portuguese population by João Maroco [26,27]. This scale consists of 15 questions and assesses three dimensions of burnout: emotional exhaustion (5 questions, corresponding to numbers 1, 4, 10, 13, 7), disbelief (4 questions, corresponding to numbers 5, 2, 11, 14), and academic efficacy (6 questions, corresponding to numbers 15, 6, 8, 12, 9, 3). In this study, the term “academic inefficacy” was used by inverting the “academic efficacy” items [27]. The MBI-SS does not allow for the calculation of an overall burnout score. High scores for emotional exhaustion (EE) and disbelief (D), along with low scores for academic efficacy (AI), were considered consistent with burnout. The 75th percentile was used as the cutoff point for each dimension [7].

### 2.6. Internal Consistency

All questionnaires used demonstrated satisfactory internal consistency results for this sample (Cronbach’s Alpha > 0.7) (Table A1) [28].

### 2.7. Statistical Analysis

Statistical analysis was conducted using IBM^®^ SPSS^®^ Statistics software, version 29. A significance level of *p* (*p*-value) < 0.05 was set with a 95% confidence interval.

The assessment of normality was conducted by analyzing skewness and kurtosis values of the distribution, which is one of the most suitable methods for larger samples (*n* > 300) [29]. The skewness and kurtosis values of the sample distribution are within the ranges of [−2, 2] and [−7, 7], respectively, indicating there is no significant deviation from a normal distribution [30]. Furthermore, according to the Central Limit Theorem, for samples larger than 30, the sampling distribution tends to resemble a normal distribution [31]. Therefore, the assumption of normality is considered to be satisfied.

The following methods were used for statistical analysis:

- Univariate analysis was employed for descriptive statistics. Categorical variables were summarized using absolute frequency (N) and percentage (%), while continuous variables were summarized using mean (M), standard deviation (SD), and confidence intervals (CI).
- Bivariate analysis using:
  ○ The chi-square test (*X*^2^) was used to compare the distribution of proportions of nominal categorical variables by sexual orientation. When more than 20% of cells had an expected count below 5, Fisher’s Exact Test results were reported. For effect size measuring, Phi coefficient (*φ*) was used when both variables were dichotomous, while Cramer’s V (*V*) was applied in all other cases.
  ○ The Mann–Whitney U test (*U*) was used to compare the differences in ordinal variable distributions by sexual orientation. The effect size was calculated and reported using *r*.
  ○ The Independent Samples *t*-test (*t*) was used to compare MSPSS, BDI, STAI-Y, and MBI means by sexual orientation. Interpretation of effect size in *t*-tests followed Cohen’s (1988) criteria [32].
- Multiple Linear Regression was performed using the General Linear Model (GLM) to explore the relationships between the predictors (Table A2) and the dependent variables BDI (depression) and STAI-Y1 (state anxiety). Only STAI-Y1 was included for anxiety, as Pearson’s correlation showed a strong link with trait anxiety (*r* = 0.90, *p* < 0.001). Interaction terms between sexual orientation and each predictor were also added. The GLM was chosen over traditional linear regression to allow flexible testing of group effects and interactions involving categorical variables [33,34]. The regression model was based on the one developed by Vânia D’Alva Teixeira [9]. Variables with no significant differences between the LGBTQ+ and heterosexual groups, specifically age, medical school, and academic year, were excluded. The main assumptions necessary to carry out multiple linear regression were met. For effect size measuring partial eta squared (*η*^2^*p*) was used [35].
  ○ Multivariate analysis was performed using the GLM Univariate framework. Specifically, we implemented a custom model structure where all independent variables were first entered to assess their main effects on psychological distress, followed by the insertion of two-way interaction terms between each variable and sexual orientation. This allowed us to explicitly test whether sexual orientation moderates the relationship between these contextual factors and students’ mental health.

The variables examined were organized as follows:

- Variables with a binary response (yes/no) were summed and recoded as “no response/no problem” (0) and “one or more responses/problems” (1).
- Likert variables were converted to a 0–100 scale, then summed and recoded into categories: “Not at all” (0), “Little” (1), “Medium” (2), and “A lot” (3).

To create the new variable “Burnout,” the three subscales representing the dimensions of Burnout were summed. Then, a new coding was done where all values below the 75th percentile were labeled as “No burnout” (0), and all values above as “Burnout” (1).

## 3. Results

### 3.1. Personal, Sociodemographic, Economic, and Academic Factors

The study included 2004 medical students from nine medical schools nationwide. The sample comprised 675 students enrolled in the 2022/2023 academic year, 599 in the 2023/2024 academic year, and 730 in the 2024/2025 academic year. The general characteristics of the sample are detailed in Table A3.

Most participants in the study were under 25 years old (87.9%), female (83.2%), and heterosexual (83.2%). The medical schools with the highest representation were the University of Minho (22%), the University of Coimbra (18.6%), and the University of Porto (17%). Most students were in their pre-clinical years (52.5%).

A statistically significant difference in sex distribution was identified between groups with different sexual orientations (*p* < 0.001, *V* = 0.21). The Heterosexual group has a higher percentage of women (86.2%), while the LGBTQ+ group has a larger share of men (27.7%) and individuals who identify as “Other” (3.87%). None of the other variables examined showed statistically significant differences between the Heterosexual and LGBTQ+ groups.

The socioeconomic analysis of the sample (Table A4) shows that most students’ parents are actively employed (>85%) and do not require social assistance grants (70.8%). Regarding parental educational attainment, both parents generally possess a higher level of education, with mothers especially likely to have one (63.2%). There was no link between socioeconomic status variables and sexual orientation.

The analysis of academic factors (Table A5) shows that the most common factor influencing course choice was “vocational interest” (83.8%). For most students, enrolling in university involved a change of residence (58.6%). A comparison between the variables listed in the table below and the variable sexual orientation indicated an association between sexual orientation and both the influencing factor in course choice (*p* < 0.001, *V* = 0.11) and the students’ cohabitation status at the time of data collection (*p* = 0.021, *V* = 0.08). No statistically significant differences were found between the groups for the other variables related to academic factors.

Regarding the reason for choosing a medical course:

- Vocational interest is the most influential factor for both groups, but it is more prevalent among heterosexuals (85.2%) than among LGBTQ+ individuals (77.1%).
- Professional and economic security is the second most important factor, but it is more common among the LGBTQ+ group (17.9%) than among heterosexuals (13.4%).
- Parental pressure is more influential among the LGBTQ+ community (4.17%) compared to the heterosexual community (1.14%).
- The influence of friends is a lesser factor but is more common among the LGBTQ+ group (0.89%) than the heterosexual group (0.40%).

Regarding students’ cohabitation status:

- Living “With my family of origin” is the most common choice for both groups, with very similar percentages.
- Living “With other family members” is more common among LGBTQ+ students (5.95%) than among heterosexuals (2.76%).
- Living in a “University residence” is less common among the LGBTQ+ group (5.06%) compared to heterosexuals (7.19%).
- Living “With other students” is an option with nearly identical percentages for both groups.

The analysis of perceived academic performance (Table A6) shows that students’ satisfaction with their grades is lower than that of their parents. However, most participants are still satisfied with their grades (61.7%).

It was found that sexual orientation is not related to perceived academic performance based on effort. However, there is a significant difference in satisfaction levels with grades, both among students (*p* < 0.001, *r* = 0.08) and in their perception of their parents’ satisfaction (*p* = 0.008, *r* = 0.06). In both cases, students and parents of heterosexual students have higher average U-test scores than those of LGBTQ+ students, suggesting they are more satisfied with their academic grades.

The analysis of perceived problems or difficulties (Table A7) shows that “Organization of Academic Work” (74.1%) is the most common problem or difficulty. “Physical Health” (49.3%), “Academic Performance” (43.6%), and “Organization of Daily Tasks” (34.7%) are other common issues among the sample. Data analysis indicates that LGBTQ+ students experience more difficulties than heterosexual students in various areas, including academic performance (*p* = 0.009, *φ* = 0.06), organization of daily tasks (*p* < 0.001, φ = 0.09), organization of academic work (*p* = 0.020, φ = 0.05), money management (*p* < 0.001, *φ* = 0.09), relationships with peers (*p* = 0.003, *φ* = 0.07), faculty (*p* = 0.002, *φ* = 0.07), and family (*p* < 0.001, *φ* = 0.10), and tobacco (*p* < 0.001, *φ* = 0.10) and other drug abuse (*p* < 0.001, *φ* = 0.11). This is a statistically significant trend in nearly all variables analyzed, except for relationship problems with a partner, physical health issues, and alcohol abuse. The percentage of heterosexual students who do not report any difficulties is higher than in the LGBTQ+ group (*p* = 0.003, *φ* = −0.07).

The analysis of negative life events in the past six months (Table A8) shows that a “serious illness/accident in someone close” is the most common negative event experienced by all students (25,2%). The data analysis shows that LGBTQ+ students face more financial difficulties (*p* = 0.004, *φ* = 0.06) and have more frequently ended stable romantic relationships (*p* = 0.011, *φ* = 0.06) in the past six months compared to heterosexual students. For other negative life events, such as the death of a family member, illness, or accident, there are no significant differences between the two groups.

The analysis of psychological support resources (Table A9) shows that most students have not sought psychological support during the medical course (55.7%). However, the majority of LGBTQ+ students have sought psychological support (62.2%).

Data analysis shows that a significantly higher percentage of LGBTQ+ students utilize mental health support resources across all categories (family doctor, psychiatrist, psychologist, and other professionals) compared to heterosexual students.

The analysis of perceived social support (Table A10) reveals that most students are satisfied with their social support network (83.18%). However, LGBTQ+ students’ U-test mean score is lower than their heterosexual peers, indicating they perceive less social support. This pattern is consistent across all indicators.

Regarding the scores on the scale that measures the perception of social support (MSPSS), it was found that LGBTQ+ students scored significantly lower on average compared to the heterosexual group, except for the MSPSS subscale that measures social support from friends (Table A11). The greatest difference between the groups is in family social support, with a moderate to large difference (*p* < 0.001, Cohen’s *d* = 0.62).

### 3.2. Prevalence and Severity of Anxiety, Depression, and Burnout Symptoms

The analysis of distress prevalence (Table A12) indicates that 43.2% of students have a BDI score suggesting depressive illness (BDI > 12). Depressive symptoms are significantly more common among LGBTQ+ students (*p* < 0.001, *r* = 0.15), found in 56.5% of this group.

Regarding anxiety, symptoms of state anxiety and trait anxiety are present in 25.7% and 26.2% of the sample, respectively. State anxiety (*p* = 0.003, *φ* = 0.07) and trait anxiety (*p* < 0.001, *φ* = 0.11) symptoms are more common among LGBTQ+ students than heterosexual students.

Regarding burnout, the prevalence of emotional exhaustion, disbelief, and academic ineffectiveness was 25.6%, 25.1%, and 25.1%. Among LGBTQ+ students, the prevalence of these burnout dimensions is higher compared to heterosexual peers, at 30.7% (*p* = 0.020, *φ* = 0.05), 30.7% (*p* = 0.010, *φ* = 0.06), and 32.1% (*p* = 0.001, *φ* = 0.07), respectively.

Similarly, the analysis of mean distress scores (Table 1) showed that LGBTQ+ students showed significantly higher levels of depression, anxiety, and burnout compared to their heterosexual peers. The biggest difference between the groups is in the Depression score, with the difference being approximately moderate (*p* < 0.001, Cohen’s *d* = −0.46).

### 3.3. Multiple Linear Regression with Interactions

#### 3.3.1. Depression

The tested model has a significant correlation (*p* < 0.001) with the dependent variable BDI (Table 2), explaining about 50% of its total variance (Adjusted R Square = 0.50). Regarding the main effects, the predictors with the greatest effect on depression levels, listed in decreasing order of partial eta squared (*η^2^p*), were burnout (B = 8.26, CI [7.31; 9.20]), low social support (B = 16.3, CI [12.5; 20.1]), low satisfaction with academic grades (B = 3.83, CI [1.57; 6.09]), female sex (B = 2.21, CI [1.17; 3.24]) compared to male sex, problems with academic performance (B = 2.20, CI [1.36; 3.04]), problems within the academic community (B = 2.27, CI [1.38; 3.16]), family and emotional issues (B = 1.95, CI [1.22; 2.67]), and substance abuse (B = 2.31, CI [0.42; 4.20]). All these factors predict higher levels of depression among medical students. The complete table regarding GLM parameter estimates is available in Table A13.

**Figure 1:**
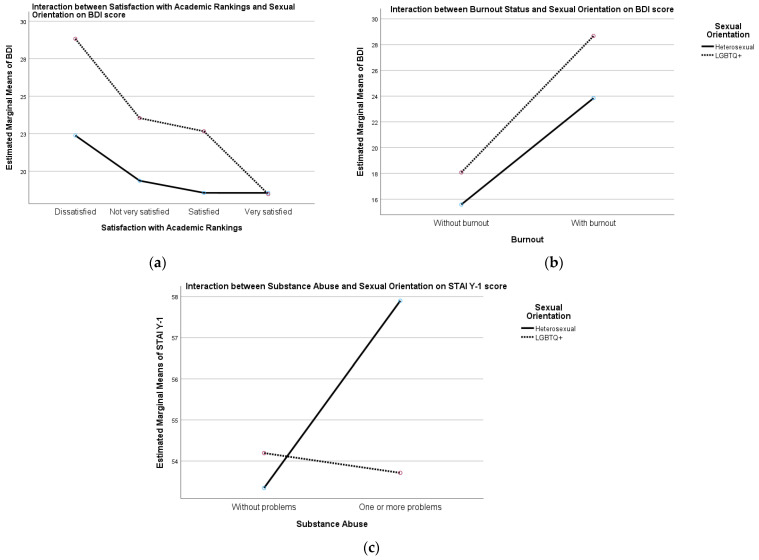
(**a**) Estimated marginal means of Beck’s Depression Inventory (BDI) based on satisfaction with academic grades, separated by sexual orientation. (**b**) Estimated marginal means of Beck’s Depression Inventory (BDI) across burnout status by sexual orientation. (**c**) Estimated marginal means of State-Trait Anxiety Inventory (STAI) across substance abuse status by sexual orientation.

Regarding the interactions between different sexual orientations and various predictors, only Satisfaction with Classifications (*p* = 0.048) and Burnout (*p* = 0.034) show a significantly different effect in each group, as illustrated in the following graphs (graphs (a) and (b) from Figure 1).

The graph (a) in Figure 1 indicates that higher satisfaction with grades is associated with lower levels of depression in both groups. However, low satisfaction with grades has a stronger impact on BDI levels for LGBTQ+ students, linking to higher depression levels.

Graph (b) in Figure 1 shows that burnout is linked to higher depression levels in both groups. However, the impact of burnout on BDI scores is stronger for LGBTQ+ students, who tend to experience greater depression.

#### 3.3.2. Anxiety

The tested model significantly correlates (*p* < 0.001) with the dependent variable STAI Y-1 (Table 3), explaining about 43% of the total variance in STAI Y-1 (adjusted R-squared = 0.43). Regarding main effects, the predictors with the greatest effect on depression levels, ranked by decreasing η^2^p, were burnout (B = 9.48, CI [8.19; 10.8]), low social support (B = 14.5, CI [9.26; 19.8]), low satisfaction with academic grades (B = 4.12, CI [1.01; 7.22]), problems in academic performance (B = 3.38, CI [2.23; 4.54]), female sex compared to male (B = 3.16, CI [1.73; 4.58]), problems in daily organization (B = 3.48, CI [2.28; 4.68]), and family and affective problems (B = 2.80, CI [1.81; 3.80]). All these predictors are linked to higher anxiety levels in medical students. The complete table regarding GLM parameter estimates is available in Table A14.

Regarding the interactions between different sexual orientations and various predictors, only substance abuse (*p* < 0.032) shows a significantly different effect in each group, as illustrated in Graph (c) in Figure 1.

The graph (c) in Figure 1 indicates that substance abuse has varying effects on STAI Y-1 levels based on sexual orientation. For heterosexual students, substance abuse issues correlate with higher levels of anxiety, while for LGBTQ+ students, substance abuse correlates with slightly lower anxiety levels.

## 4. Discussion

The prevalence of depression among medical students found in this study is higher than the rates reported in both national and international analyses, even when using the same scale [3,7,36,37,38]. These analyses predate 2020 and the current study, which may reflect the effects of the COVID-19 pandemic, associated not only with higher depression levels but also with increased awareness and reduced stigma about mental illness, potentially leading to more reporting of symptoms [39,40]. Regarding the prevalence of anxiety, the rates are similar to those reported in other studies [36,37,41]. Burnout appears less common in this study compared to others, possibly because of the higher cutoff level used here [42,43,44]. High levels of stress among medical students are commonly reported in the literature [7,45,46].

This study aims to add to existing knowledge by focusing on a specific group of medical students: LGBTQ+ students. The main goal is to test the hypothesis that distress levels differ based on sexual orientation. To our knowledge, this is the first study in Portugal to directly compare medical students of different sexual orientations across multiple domains (personal, social, academic, family, financial, and psychiatric). The analysis used a large national sample that included students from nine medical schools across the country. Levels of anxiety, depression, and burnout were measured with validated scales that enable comparison with previous research. Additionally, a regression analysis with interaction terms was conducted, providing results that deepen the understanding of how predictors associate with distress across different sexual orientations.

The sample consists of 1668 (83.2%) heterosexual students and 336 (16.8%) LGBTQ+ students, with the proportion of the latter group falling within the 5–19% range reported by similar international studies [12,14,16,17]. However, this proportion may be inaccurate and lower than the actual value, as there could be underreporting of minority sexual orientations due to stigma.

The results support the hypothesis that, in Portugal, the LGBTQ+ community experiences a higher prevalence and severity of depression, anxiety, and burnout compared to heterosexual students. This finding is also supported by international studies [11,12,14,15,16,17]. However, when examining sexual orientation while adjusting for other predictors, it was found that other factors predict higher levels of distress than sexual orientation. This may suggest that the increased vulnerability seen in LGBTQ+ students is not directly caused by sexual orientation itself but is more likely due to greater exposure to additional vulnerability factors, such as increased personal, academic, social, and financial difficulties, as observed in this study and others [11,17].

However, there are variables identified in international studies that were not explored in this study, and that may constitute unique risk factors for LGBTQ+ students, such as dissatisfaction or discomfort with gender identity or sexual orientation, the high incidence of stigma and discrimination against LGBTQ+ students, and a non-inclusive academic environment [11,12,13,14,15,16,17].

When interpreting these findings, the evaluation of effect sizes provides critical insight into the practical reality of LGBTQ+ medical students in Portugal. The medium effect size observed for depression (d = −0.46) is particularly alarming, indicating that the disparity in depressive symptomatology is not merely a statistical artifact of our large sample size (N = 2004), but a robust, clinically meaningful divergence [32,35]. Conversely, while the effect sizes for anxiety and burnout dimensions were smaller, they must not be dismissed. In the highly pressurized and homogenous environment of medical training, even a ‘small’ structural shift in emotional exhaustion or academic disbelief can be the tipping point that leads to academic attrition or severe psychological crises. These effect sizes reinforce that sexual orientation acts as a distinct, independent source of minority stress within medical faculties.

Burnout was the main factor associated with higher depression and anxiety levels, consistent with earlier research [7,47]. Furthermore, burnout was found to be linked with higher levels of depression among LGBTQ+ students, and this connection was also identified in other studies [12]. In other words, the impact of emotional exhaustion, disbelief, and academic ineffectiveness is even more harmful for the LGBTQ+ group. This result may be because this group not only has less social support but also faces more challenges in academic, family, and emotional areas. These findings emphasize the importance of prioritizing students’ mental health in medical training, regularly addressing this issue throughout medical education, and adjusting the academic workload.

Another predictor that is significantly associated with distress levels is social support, with less social support linked to higher depression and anxiety. This finding aligns with earlier research [48,49]. Overall, the sample shows high levels of social support, but the LGBTQ+ students report lower levels compared to heterosexual students, which aligns with international research [11,14,16,17]. This is concerning since having a strong social support network and satisfaction with social activities are known as protective factors and coping tools for managing academic challenges and preventing distress, especially among LGBTQ+ students [9,11,50]. This lower level of social support among LGBTQ+ individuals may stem from increased family problems, conflicts with academic community members, and other factors like past discrimination and non-inclusive academic environments [14,16,17]. Therefore, it is crucial for both universities and medical schools to actively create policies promoting inclusion, visibility, and normalization of diversity to foster a more welcoming academic environment.

Next, academic factors such as satisfaction with academic grades and academic difficulties had the greatest impact on anxiety and depression levels, with low satisfaction with academic results and academic difficulties associated with higher levels of distress. This finding aligns with the results of previous analyses [47]. Understandably, dissatisfaction with academic performance can become more than just an academic issue, significantly affecting personal validation [51].

This issue is even more prominent among the LGBTQ+ group; these students are less satisfied with their grades and report more academic challenges than heterosexual students, which aligns with other studies [11]. The greater impact on this group can be attributed to a stronger need for recognition, as social support is weaker, and other challenges are more prevalent across various areas. It may also be linked to the higher number of LGBTQ+ students who choose Medicine for “professional and financial security.” Therefore, it is crucial to provide students with an environment where they can make mistakes and explore different aspects of their personal and academic identities.

In the personal dimension, being female was a predictor of higher levels of distress. This relationship has already been documented in the literature [9]. This result may be due to multiple factors, such as the higher prevalence of personality traits linked to increased perfectionism and impostor syndrome in female students [52]. Furthermore, female medical students often experience negative life events more intensely and also bear the burden of gender expectations [53]. Addressing distress in female students can be achieved through programs that focus on developing coping strategies. The effect of sex on distress was found to be independent of sexual orientation. It was not possible to determine links between distress and non-binary and transgender individuals because of their low representation in the sample.

The sample’s gender profile must be considered in this study, as the high percentage of women (83.2%) reflects the current reality of Portuguese medical schools [7,9]. However, the literature shows that women generally report higher levels of anxiety and depression [8,47]. This high proportion may have increased the overall distress scores of the study. Additionally, the percentage of women varied across sexual orientation groups. This imbalance could act as a confounding variable. To resolve this and isolate the true association of sexual orientation with distress, sex was strictly controlled as a fixed factor in our multivariate analysis. Future studies with balanced samples should further explore the intersection between sex and sexual orientation.

At the family and relationship levels, problems with family and partners predict distress, and their effects are consistent regardless of sexual orientation. However, LGBTQ+ students more often face relationship issues with family, which aligns with findings from other studies [14]. This may be because the families of LGBTQ+ students often hold conservative expectations tied to heteronormativity, which can lead to family conflict and result in less social support.

Substance abuse was also identified as a predictor of higher depression levels, but it was not a significant predictor of anxiety. LGBTQ+ students reported greater tobacco and other drug use compared to heterosexual students, with no differences in alcohol consumption. This finding aligns with other studies conducted with LGBTQ+ individuals [54]. However, substance abuse correlates with anxiety levels differently based on sexual orientation. For heterosexual students, substance abuse is linked to higher anxiety, likely due to guilt and stress caused by the abuse, which may increase the risk of developing anxiety. For LGBTQ+ students, substance abuse is linked to lower anxiety levels, and the substances most often used by this group may serve as a coping mechanism for issues across multiple areas, though this is an unhealthy and harmful way of coping.

The elevated rates of depression, anxiety, and burnout observed among LGBTQ+ medical students in our sample cannot be viewed merely as individual psychological vulnerabilities; rather, they reflect the systemic mechanisms outlined in Minority Stress Theory [10]. LGBTQ+ students face a ‘double burden’: the baseline, rigorous academic demands of medical training shared by all peers, compounded by proximal and distal minority stressors—such as microaggressions, hypervigilance regarding identity disclosure, and a heteronormative institutional climate.

This framework helps explain why academic satisfaction and social support emerged as such powerful determinants in our study. For a minority student, low academic satisfaction is rarely just about a difficult curriculum; it often stems from a lack of inclusive representation, biased clinical interactions, or a feeling of institutional alienation. Conversely, robust social support does not merely offer general comfort; it acts as a critical structural buffer that validates the student’s marginalized identity against a heteronormative environment. When these buffers are weak or absent, the compounding effect of minority stress accelerates emotional exhaustion, explaining why variables like burnout carry a disproportionately severe impact on the mental health trajectories of LGBTQ+ future physicians.

To reduce distress among LGBTQ+ medical students, structural and policy changes are needed. In Portugal, despite advanced national legislation—such as the National Strategy for Equality and Non-Discrimination (ENIND)—the practical implementation of these policies in medical schools remains limited [55].

To address this, institutions should adopt evidence-based interventions such as specific LGBTQ+ healthcare competencies in the formal medical curriculum, creating confidential institutional support spaces and mandatory faculty training on microaggressions, and establishing formal peer-mentorship networks. Implementing these models is crucial to providing structural social support, reducing burnout, and fostering a safer academic environment [56,57].

This study has several limitations, such as its observational and cross-sectional design, which prevents establishing causality. Additionally, using a convenience sample may not accurately represent Portuguese medical students. It is possible that more responses came from individuals more interested in the topic being studied (e.g., students experiencing distress symptoms), which could lead to an overestimation of the results. The self-report nature of the questionnaires is another limitation, as it prevents reliable identification of the number of people diagnosed with depressive or anxiety disorders. Comparing groups with different sample sizes may also pose a limitation. Finally, the limited literature on distress in the Portuguese LGBTQ+ medical student population restricts the number of comparisons and the framing of the results.

## 5. Conclusions

This study found that the rates and severity of depression, anxiety, and burnout symptoms among medical students are high, with variations based on sexual orientation. LGBTQ+ students not only face higher levels of distress than heterosexual students but also report more challenges in personal, academic, social, and financial areas. These findings suggest that academic institutions should develop inclusive institutional policies, and the promotion of safe and non-discriminatory learning environments may contribute to reducing minority stress among LGBTQ+ students. Medical schools and policymakers should also consider implementing structured well-being programs integrated into the medical curriculum, aimed at early identification and prevention of psychological distress.

## Figures and Tables

**Table 1:** Descriptive analysis of distress prevalence and comparison of frequency distribution by sexual orientation.

Levels of Distress	All (N = 2004)		Heterosexual (N = 1668)		LGBTQ+ (N = 336)		*t*	*p*	*d*
	M ± SD	CI	M ± SD	CI	M ± SD	CI			
Depression	13.1 ± 10.3	[12.7; 13.6]	12.4 ± 9.71	[11.9; 12.8]	17.0 ± 12.0	[15.7; 18.3]	−6.64	**<0.001**	−0.46
State Anxiety	46.8 ± 13.3	[46.2; 47.4]	46.3 ± 13.2	[45.7; 46.9]	49.1 ± 13.3	[47.7; 50.6]	−3.59	**<0.001**	−0.22
Trait Anxiety	47.0 ± 13.7	[46.4; 47.6]	46.2 ± 13.5	[45.5; 46.8]	51.0 ± 13.8	[49.5; 52.4]	−5.91	**<0.001**	−0.35
Emotional Exhaustion	16.6 ± 6.58	[16.3; 16.9]	16.4 ± 6.53	[16.0; 16.7]	17.8 ± 6.74	[17.0; 18.5]	−3.62	**<0.001**	−0.22
Disbelief	8.80 ± 5.69	[8.55; 9.05]	8.60 ± 5.60	[8.33; 8.87]	9.82 ± 6.04	[9.18; 10.5]	−3.62	**<0.001**	−0.22
Academic Ineffectiveness	15.5 ± 5.75	[15.2; 15.7]	15.3 ± 5.64	[15.0; 15.5]	16.5 ± 6.18	[15.9; 17.2]	−3.64	**<0.001**	−0.22

Statistically significant values are in bold. N: Absolute Frequency; M: Mean; SD: Standard deviation; CI: Confidence intervals; *t*: *t* test; *p*: *p*-value; *d*: Cohen’s *d*.

**Table 2:** Values Related to the Linear Regression for the Dependent Variable Depression.

Main Effects and Interactions (GLM) Analysis in the Prediction of Depression (BDI)	*F*	*p*	*η* ^2^ *p*
Sexual Orientation	3.67	0.056	0.00
Sex	5.89	**0.003**	0.01
Substance Abuse	6.75	**0.009**	0.00
Satisfaction with Academic Rankings	10.9	**<0.001**	0.02
Problems associated with Academic Performance	10.3	**0.001**	0.01
Problems associated with Daily Organization	3.55	0.060	0.00
Problems in Academic Community	22.1	**<0.001**	0.01
Family and Affective Problems	10.8	**0.001**	0.01
Financial Problems	0.37	0.544	0.00
Social Support	44.7	**<0.001**	0.06
Burnout	295	**<0.001**	0.13
Sexual Orientation * Sex	0.80	0.448	0.00
Sexual Orientation * Substance Abuse	0.01	0.913	0.00
Sexual Orientation * Satisfaction with Academic Rankings	2.64	**0.048**	0.00
Sexual Orientation * Problems associated with Academic Performance	0.51	0.475	0.00
Sexual Orientation * Problems associated with Daily Organization	0.33	0.564	0.00
Sexual Orientation * Problems in Academic Community	0.16	0.689	0.00
Sexual Orientation * Family and Affective Problems	0.85	0.356	0.00
Sexual Orientation * Financial Problems	0.40	0.527	0.00
Sexual Orientation * Social Support	0.39	0.759	0.00
Sexual Orientation * Burnout	4.52	**0.034**	0.00

R Square = 0.50 (Adjusted R Square = 0.50). Statistically significant values are in bold. GLM: General linear model; BDI: Beck’s Depression Inventory; *F*: Anova’s F; *p*: *p*-value; *η*^2^*p*: Partial eta squared. *: Represents interactions between variables.

**Table 3:** Values related to the linear regression for the dependent variable anxiety.

Main Effects and Interactions (GLM) Analysis in the Prediction of Anxiety (STAI Y-1)	*F*	*p*	*η* ^2^ *p*
Sexual Orientation	0.40	0.526	0.00
Sex	4.39	**0.013**	0.00
Substance Abuse	3.01	0.083	0.00
Satisfaction with Academic Rankings	6.36	**<0.001**	0.01
Problems associated with Academic Performance	10.2	**0.001**	0.01
Problems associated with Daily Organization	5.38	**0.021**	0.00
Problems in Academic Community	3.54	0.060	0.00
Family and Affective Problems	6.59	**0.010**	0.00
Financial Problems	2.60	0.107	0.00
Social Support	35.0	**<0.001**	0.05
Burnout	184	**<0.001**	0.09
Sexual Orientation * Sex	1.61	0.200	0.00
Sexual Orientation * Substance Abuse	4.60	**0.032**	0.00
Sexual Orientation * Satisfaction with Academic Rankings	1.32	0.267	0.00
Sexual Orientation * Problems associated with Academic Performance	1.39	0.239	0.00
Sexual Orientation * Problems associated with Daily Organization	2.77	0.096	0.00
Sexual Orientation * Problems in Academic Community	2.35	0.126	0.00
Sexual Orientation * Family and Affective Problems	3.34	0.068	0.00
Sexual Orientation * Financial Problems	0.06	0.808	0.00
Sexual Orientation * Social Support	0.33	0.806	0.00
Sexual Orientation * Burnout	1.03	0.311	0.00

R Square = 0.44 (Adjusted R Square = 0.43). Statistically significant values are in bold. GLM: General linear model; BDI: Beck’s Depression Inventory; *F*: Anova’s F; *p*: *p*-value; *η*^2^*p*: Partial eta squared. *: Represents interactions between variables.

## Data Availability

The original contributions presented in the study are included in the article, further inquiries can be directed to the corresponding author.

## Abbreviations

The following abbreviations are used in this manuscript:

BDI	Beck’s Depression Inventory
CI	Confidence Interval
GLM	General Linear Model
MBI-SS	Maslach Burnout Inventory—Student Survey
MSPSS	Multidimensional Scale of Perceived Social Support
*p*	*p*-value (level of statistical significance)
η^2^p	Partial Eta Squared
φ	Phi coefficient
STAI	State-Trait Anxiety Inventory—Form Y
V	Cramer’s V

## Appendix A

**Table A1:** Internal consistency of the assessment instruments used.

Instrument	Cronbach’s Alpha
MSPSS (Family)	0.92
MSPSS (Friends)	0.95
MSPSS (Other)	0.93
MSPSS (Total)	0.92
BDI	0.92
STAI Y-1 (State anxiety)	0.95
STAI Y-2 (Trait anxiety)	0.95
MBI-SS (Emotional exhaustion)	0.89
MBI-SS (Disbelief)	0.84
MBI-SS (Academic inefficacy)	0.79

**Table A2:** Description of the Independent Variables used in General Linear Model.

Model	Independent Variable Created	Grouped Independent Variables
Personal Factors	Sexual Orientation	
	Sex	
	Substance Abuse	Substance Abuse: Alcohol
		Substance Abuse: Tobacco
		Substance Abuse: Other drugs
Academic Factors	Satisfaction with Academic Rankings	
	Problems associated with Academic Performance	
	Problems associated with Daily Organization	Problems related to the Organization of Daily Tasks
		Problems related to the Organization of Academic Work
	Problems in Academic Community	Problems in Relationships with Colleagues
		Problems in the Relationship with Professors
Social and Financial Factors	Family and Affective Problems	Problems in Family Relationships
		Problems in Boyfriend/Girlfriend Relationships
		Death of a Family Member in the Last Six Months
		End of a Stable Love Relationship in the Last Six Months
		Someone Important Has Suffered an Illness or Accident in the Last Six Months
	Financial Problems	Difficulties Associated with Money Management
		Financial Problems in the Last Six Months
	Social Support	Number of Close People
		Involvement and Interest of Close People
		Level of Satisfaction with Social Activities
		Level of Satisfaction with Support of the Social Relations Network
Distress Factors	Burnout	Emotional Exhaustion
		Disbelief
		Academic Ineffectiveness

**Table A3:** General overview of the sample and comparison of frequency distribution by sexual orientation.

General Characterization of the Sample	All (N = 2004)		Heterosexual (N = 1668)		LGBTQ+ (N = 336)		Statistical Test		
	**N**	**%**	**N**	**%**	**N**	**%**	***X*** **^2^** **/*U***	***p***	***φ*** **/*V*/*r***
**Academic Year**							279,629	0.948	<0.01
2022–2023	675	33.7	559	33.5	116	34.5			
2023–2024	599	29.9	505	30.3	94	28.0			
2024–2025	730	36.4	604	36.2	126	37.5			
**Age**							274,406	0.545	0.01
17	51	2.54	43	2.58	8	2.38			
18	272	13.6	238	14.3	34	10.1			
19	274	13.7	215	12.9	59	17.6			
20	251	12.5	205	12.3	46	13.7			
21	258	12.9	214	12.8	44	13.1			
22	252	12.6	204	12.2	48	14.3			
23	253	12.6	212	12.7	41	12.2			
24	151	7.53	128	7.67	23	6.85			
≥25	242	12.1	209	12.5	33	9.82			
**Sex**							89.1	**<0.001**	0.21
Female	1668	83.2	1438	86.2	230	68.5			
Male	319	15.9	226	13.5	93	27.7			
Other	17	0.85	4	0.20	13	3.87			
**Medical School**							10.8	0.215	0.07
UM	441	22.0	370	22.2	71	21.1			
UNL	138	6.89	109	6.53	29	8.63			
UBI	137	6.84	115	6.89	22	6.55			
UAL	74	3.69	68	4.08	6	1.79			
UL	327	16.3	259	15.5	68	20.2			
UC	373	18.6	315	18.9	58	17.3			
FMUP	341	17.0	288	17.3	53	15.8			
ICBAS	106	5.29	87	5.22	19	5.65			
UCP	67	3.34	57	3.42	10	2.98			
**Curricular Year**							278,983	0.896	<0.01
1st year	418	20.9	355	21.3	63	18.8			
2nd year	376	18.8	300	18.0	76	22.6			
3rd year	258	12.9	216	12.9	42	12.5			
4th year	262	13.1	220	13.2	42	12.5			
5th year	286	14.3	240	14.4	46	13.7			
6th year	404	20.2	337	20.2	67	19.9			
**Medical Training Phase**							275,598	0.580	0.01
Preclinical Years	1052	52.5	871	52.2	181	53.9			
Clinical Years	952	47.5	797	47.8	155	46.1			

Statistically significant values are in bold. N: Absolute Frequency; *X*^2^: Chi-square test; *U*: Mann–Whitney’s U; *p*: *p*-value; *φ*: Phi coefficient; *V*: Cramer’s V; *r*: r-value.

**Table A4:** Descriptive analysis of the socioeconomic situation and comparison of the frequency distribution by sexual orientation.

Socioeconomic Situation	All (N = 2004)		Heterosexual (N = 1668)		LGBTQ+ (N = 336)		Statistical Test		
	**N**	**%**	**N**	**%**	**N**	**%**	***X*** **^2^** **/*U***	***p***	***φ*** **/*V*/*r***
**Scholarship Holder Status**							2.07	0.355	0.032
I need a scholarship, but I didn’t get it	212	10.6	182	10.9	30	8.93			
I have a scholarship	374	18.7	304	18.2	70	20.8			
I am not a scholarship holder, nor do I need it	1418	70.8	1182	70.9	236	70.2			
**Father’s Professional Status**							6.24	0.101	0.056
Active	1730	86.3	1436	86.1	294	87.5			
Unemployed	48	2.40	35	2.10	13	3.87			
Retired	178	8.88	155	9.29	23	6.85			
Deceased	48	2.40	42	2.52	6	1.79			
**Mother’s Professional Status**							4.62	0.202	0.048
Active	1742	86.9	1452	87.1	290	86.3			
Unemployed	163	8.13	129	7.73	34	10.1			
Retired	79	3.94	71	4.26	8	2.38			
Deceased	20	1.00	16	0.96	4	1.19			
**Father’s Literary Qualifications**							278,746	0.875	<0.01
No Qualifications	3	0.15	3	0.18	0	0.00			
1st Cycle of Basic Education (4th Grade)	81	4.04	68	4.08	13	3.87			
2nd Cycle of Basic Education (6th year)	152	7.58	124	7.43	28	8.33			
3rd Cycle of Basic Education (9th year)	220	11.0	177	10.6	43	12.8			
Secondary Education (12th grade)	475	23.7	400	24.0	75	22.3			
Undergraduate Education	653	32.6	552	33.1	101	30.1			
Postgraduate Education (Master’s/PhD)	412	20.6	337	20.2	75	22.3			
Unknown	8	0.40	7	0.42	1	0.30			
**Mother’s Literary Qualifications**							280,152	0.994	<0.01
No Qualifications	0	0.00	0	0.00	0	0.00			
1st Cycle of Basic Education (4th Grade)	48	2.40	42	2.50	6	1.80			
2nd Cycle of Basic Education (6th year)	104	5.19	87	5.20	17	5.10			
3rd Cycle of Basic Education (9th year)	175	8.73	141	8.50	34	10.1			
Secondary Education (12th grade)	410	20.5	339	20.3	71	21.1			
Undergraduate Education	806	40.2	680	40.8	126	37.5			
Postgraduate Education (Master’s/PhD)	459	22.9	377	22.6	82	24.4			
Unknown	2	0.10	2	0.10	0	0.00			

N: Absolute Frequency; *X*^2^: Chi-square test; *U*: Mann-Whitney’s U; *p*: *p*-value; *φ*: Phi coefficient; *V*: Cramer’s V; *r*: r-value.

**Table A5:** Descriptive analysis of academic factors and comparison of frequency distribution by sexual orientation.

Academic Factors	All (N = 2004)		Heterosexual (N = 1668)		LGBTQ+ (N = 336)		Statistical Test		
	**N**	**%**	**N**	**%**	**N**	**%**	***X*** **^2^**	***p***	***φ*** **/*V***
**Influencing Factor of Choice**							24.2	**<0.001**	0.11
Vocational Interest	1680	83.8	1421	85.2	259	77.1			
Professional and Economic Security	283	14.1	223	13.4	60	17.9			
Parental Pressure	33	1.65	19	1.14	14	4.17			
Influence of Friends	8	0.40	5	0.30	3	0.89			
**Entering university involved**									
**changing residence**							1.79	0.181	−0.03
Yes	1175	58.6	989	59.3	186	55.4			
No	829	41.4	679	40.7	150	44.6			
**Who do you live with right now**							13.3	**0.021**	0.08
With my family of origin	831	41.5	690	41.4	141	42.0			
With other family members	66	3.29	46	2.76	20	5.95			
In a university residence	137	6.84	120	7.19	17	5.06			
With other students	749	37.4	631	37.8	118	35.1			
Alone	142	7.09	120	7.19	22	6.55			
Other	79	3.94	61	3.66	18	5.36			

Statistically significant values are in bold. N: Absolute Frequency; *X*^2^: Chi-square test; *p*: *p*-value; *φ*: Phi coefficient; *V*: Cramer’s V.

**Table A6:** Descriptive analysis of perceptions of academic performance and comparison of frequency distribution by sexual orientation.

Perception of Academic Performance	All (N = 2004)		Heterosexual (N = 1668)		LGBTQ+ (N = 336)		Statistical Test		
	**N**	**%**	**N**	**%**	**N**	**%**	***U***	***p***	***r***
**Academic ratings in**							271,648	0.306	0.02
**relation to Effort Expended**									
Lower to effort expended	802	40.0	670	40.2	132	39.3			
In agreement with the effort expended	1144	57.1	960	57.6	184	54.8			
Superior to the effort expended	58	2.89	38	2.28	20	5.95			
**Level of Satisfaction with**							249,813	**<0.001**	0.08
**Academic Rankings**									
Dissatisfied	106	5.29	75	4.5	31	9.23			
Not very satisfied	661	33.0	533	32.0	128	38.1			
Satisfied	1081	53.9	932	55.9	149	44.3			
Very satisfied	156	7.78	128	7.67	28	8.33			
**Parental Satisfaction Level**							257,026	**0.008**	0.06
**regarding Academic Rankings**									
Dissatisfied	34	1.7	19	1.14	15	4.46			
Not very satisfied	144	7.19	107	6.41	37	11.0			
Satisfied	877	43.8	740	44.4	137	40.8			
Very satisfied	949	47.4	802	48.1	147	43.8			

Statistically significant values are in bold. N: Absolute Frequency; *U*: Mann–Whitney’s U; *p*: *p*-value; *r*: r-value.

**Table A7:** Descriptive analysis of the perception of problems or difficulties and comparison of the frequency distribution by sexual orientation.

Perception of Problems and Difficulties	All (N = 2004)		Heterosexual (N = 1668)		LGBTQ+ (N = 336)		Statistical Test		
	**N**	**%**	**N**	**%**	**N**	**%**	***X*** **^2^**	***p***	**φ**
Academic Performance	873	43.6	705	42.3	168	50.0	6.80	**0.009**	0.058
Organization of Daily Tasks	695	34.7	547	32.8	148	44.0	15.6	**<0.001**	0.088
Organization of Academic Work	1485	74.1	1219	73.1	266	79.2	5.40	**0.02**	0.052
Money Management	663	33.1	522	31.3	141	42.0	14.4	**<0.001**	0.085
Relationship with Colleagues	487	24.3	384	23.0	103	30.7	8.86	**0.003**	0.066
Relationship with Professors	82	4.10	58	3.48	24	7.14	9.58	**0.002**	0.069
Relationship with Family	362	18.1	272	16.3	90	26.8	20.7	**<0.001**	0.102
Relationship with boyfriend/girlfriend	139	6.90	116	6.95	23	6.85	0.005	0.943	−0.01
Physical Health	987	49.3	808	48.4	179	53.3	2.61	0.106	0.036
Substance Abuse: Alcohol	24	1.20	18	1.08	6	1.79	1.18	0.277	0.024
Substance Abuse: Tobacco	69	3.40	44	2.64	25	7.44	19.4	**<0.001**	0.098
Substance Abuse: Other drugs	19	0.93	8	0.48	11	3.27	23.3	**<0.001**	0.108
No Perception of Difficulties	149	7.44	137	8.21	12	3.57	8.76	**0.003**	−0.07

Statistically significant values are in bold. N: Absolute Frequency; *X*^2^: Chi-square test; *p*: *p*-value; *φ*: Phi coefficient.

**Table A8:** Descriptive analysis of negative life events in the past 6 months and comparison of frequency distribution by sexual orientation.

Negative Life Events in the Last Six Months	All (N = 2004)		Heterosexual (N = 1668)		LGBTQ+ (N = 336)		Statistical Test		
	**N**	**%**	**N**	**%**	**N**	**%**	***X* ^2^**	***p***	***φ***
Death of a Family Member	354	17.7	303	18.2	51	15.2	1.72	0.19	0.029
End of a Stable Love Relationship	222	11.1	171	10.3	51	15.2	6.89	**0.011**	0.059
Financial Problems	366	18.3	286	17.1	80	23.8	8.32	**0.004**	0.064
Had a Serious Illness or an Accident	92	4.59	73	4.38	19	5.65	1.04	0.307	0.023
Someone Important has suffered a serious illness	506	25.2	423	25.4	83	24.7	0.06	0.800	−0.01
or an Accident									

Statistically significant values are in bold. N: Absolute Frequency; *X*^2^: Chi-square test; *p*: *p*-value; *φ*: Phi coefficient.

**Table A9:** Descriptive analysis of psychological support resources and comparison of frequency distribution by sexual orientation.

Use of Psychological Support Resources	All (N = 2004)		Heterosexual (N = 1668)		LGBTQ+ (N = 336)		Statistical Test		
	**N**	**%**	**N**	**%**	**N**	**%**	***X*** **^2^**	***p***	***φ***
Family Doctor	211	10.5	162	9.71	49	14.6	7.04	**0.008**	0.059
Psychiatrist	351	17.5	247	14.8	104	31.0	50.5	**<0.001**	0.159
Psychologist	746	37.2	574	34.4	172	51.2	33.7	**<0.001**	0.130
Other Mental Health Support Professional	42	2.10	28	1.68	14	4.17	8.44	**0.004**	0.065
Did not Use of any of these Resources	1116	55.7	989	59.3	127	37.8	52.4	**<0.001**	−0.16

Statistically significant values are in bold. N: Absolute Frequency; *X*^2^: Chi-square test; *p*: *p*-value; *φ*: Phi coefficient.

**Table A10:** Descriptive analysis of perceived social support and comparison of frequency distribution by sexual orientation.

**Perception of Social Support**	**All** **(N = 2004)**		**Heterosexual** **(N = 1668)**		**LGBTQ+** **(N = 336)**		**Statistical Test**		
	**N**	**%**	**N**	**%**	**N**	**%**	***U***	***p***	***r***
**Number of Close People**							255,605	**0.006**	0.06
**They Trust**									
None	22	1.10	14	0.84	8	2.38			
1 or 2	516	25.7	411	24.6	105	31.3			
3 to 5	906	45.2	767	46.0	139	41.4			
More than 5	560	27.9	476	28.5	84	25.0			
**Interest and Involvement**							247,025	**<0.001**	0.09
**of Close People**									
None	11	0.55	8	0.48	3	0.89			
Reduced	65	3.24	45	2.70	20	5.95			
Not Sure	107	5.34	87	5.22	20	5.95			
Some	706	35.2	570	34.2	136	40.5			
A Lot	1115	55.6	958	57.4	157	46.7			
**Level of Satisfaction with Support**							255,598	**0.005**	0.06
**of the Social Relations Network**									
Dissatisfied	44	2.20	35	2.10	9	2.68			
Not very satisfied	280	14.0	220	13.2	60	17.9			
Satisfied	1034	51.6	858	51.4	176	52.4			
Very satisfied	646	32.2	555	33.3	91	27.1			
**Level of Satisfaction with**							258,087	**0.013**	0.06
**Social Activities**									
Dissatisfied	89	4.44	73	4.38	16	4.76			
Not very satisfied	529	26.4	421	25.2	108	32.1			
Satisfied	1031	51.4	870	52.2	161	47.9			
Very satisfied	355	17.7	304	18.2	51	15.2			

Statistically significant values are in bold. N: Absolute Frequency; *U*: Mann-Whitney’s U; *p*: *p*-value; *r*: r-value.

**Table A11:** Descriptive analysis of average levels of social support and comparison by sexual orientation.

Social Support	All (N = 2004)		Heterosexual (N = 1668)		LGBTQ+ (N = 336)		*t*	*p*	*d*
	**M ± SD**	**CI**	**M ± SD**	**CI**	**M ± SD**	**CI**			
**MSPSS**									
MSPSS—Family	5.81 ± 1.29	[5.75; 5.87]	5.94 ± 1.22	[5.88; 6.00]	5.16 ± 1.38	[5.01; 5.31]	9.59	**<0.001**	0.62
MSPSS—Friends	5.75 ± 1.26	[5.70; 5.81]	5.78 ± 1.24	[5.72; 5.83]	5.63 ± 1.37	[5.49; 5.78]	1.89	0.059	0.11
MSPSS—Others	5.94 ± 1.41	[5.88; 6.00]	6.00 ± 1.36	[5.93; 6.06]	5.65 ± 1.63	[5.48; 5.83}	3.63	**<0.001**	0.25
MSPSS—Total	5.83 ± 1.05	[5.79; 5.88]	5.90 ± 1.02	[5.85; 5.95]	5.48 ± 1.10	[5.36; 5.60]	6.50	**<0.001**	0.41

Statistically significant values are in bold. N: Absolute Frequency; M: Mean; SD: Standard Deviation; CI: Confidence Intervals; *t*: *t* test; *p*: *p*-value; *d*: Cohen’s d.

**Table A12:** Descriptive analysis of average distress levels and comparison by sexual orientation.

Prevalence of Distress	All (N = 2004)		Heterosexual (N = 1668)		LGBTQ+ (N = 336)		Statistical Test		
	**N**	**%**	**N**	**%**	**N**	**%**	***X*** **^2^** **/*U***	***p***	***φ*** **/*r***
**Depression**							215,464	**<0.001**	0.146
No Depression (0 to 12)	1139	56.8	993	59.5	146	43.5			
Mild (13 to 18)	384	19.2	322	19.3	62	18.5			
Moderate (19 to 23)	240	12.0	184	11.0	56	16.7			
Severe (≥24)	241	12.0	169	10.1	72	21.4			
**Anxiety (Cut-off p75)**									
State Anxiety	515	25.7	407	24.4	108	32.1	8.78	**0.003**	0.066
Trait Anxiety	526	26.2	401	24.0	125	37.2	25.0	**<0.001**	0.112
**Burnout (Cut-off p75)**									
Emotional Exhaustion	513	25.6	410	24.6	103	30.7	5.42	**0.020**	0.052
Disbelief	504	25.1	401	24.0	103	30.7	6.50	**0.011**	0.057
Academic Ineffectiveness	504	25.1	396	23.7	108	32.1	10.4	**0.001**	0.072

Statistically significant values are in bold. N: Absolute Frequency; *X*^2^: Chi-square test; *U*: Mann–Whitney’s U; *p*: *p*-value; *φ*: Phi coefficient; *r*: r-value.

**Table A13:** Parameter estimates from depression’s general linear model analysis.

Predictor	Parameter	B	SD	*p*	CI
Sexual Orientation	LGBTQ+	−0.43	1.90	0.820	[−4.15; 3.29]
Sex	Other	−0.27	3.70	0.943	[−7.52; 6.99]
	Female	2.21	0.53	**<0.001**	[1.17; 3.24]
Substance Abuse	One or more problems	2.31	0.96	**0.016**	[0.42; 4.20]
Satisfaction with Academic Rankings	Not at all	3.83	1.15	**0.001**	[1.57; 6.09]
	Little	0.81	0.77	0.292	[−0.70; 2.32]
	Medium	0.00	0.70	0.997	[−1.36; 1.37]
Problems associated with Academic Performance	One or more problems	2.20	0.43	**<0.001**	[1.36; 3.04]
Problems associated with Daily Organization	One or more problems	1.56	0.45	**<0.001**	[0.69; 2.44]
Problems in Academic Community	One or more problems	2.27	0.45	**<0.001**	[1.38; 3.16]
Family and Affective Problems	One or more problems	1.95	0.37	**<0.001**	[1.22; 2.67]
Financial Problems	One or more problems	0.58	0.39	0.136	[−0.18; 1.33]
Social Support	Not at all	16.3	1.95	**<0.001**	[12.5; 20.1]
	Little	6.96	0.67	**<0.001**	[5.64; 8.28]
	Medium	2.78	0.40	**<0.001**	[1.99; 3.56]
Burnout	With burnout	8.26	0.48	**<0.001**	[7.31; 9.20]
Sexual Orientation * Sex	LGBTQ+ * Other	3.98	4.31	0.355	[−4.47; 12.4]
	LGBTQ+ * Female	−0.73	1.08	0.500	[−2.84; 1.38]
Sexual Orientation * Substance Abuse	LGBTQ+ * One or more problems	−0.19	1.71	0.913	[−3.53; 3.16]
Sexual Orientation * Satisfaction with Academic Rankings	LGBTQ+ * Not at all	6.54	2.55	**0.010**	[1.54; 11.5]
	LGBTQ+ * Little	4.26	1.92	**0.026**	[0.45; 8.02]
	LGBTQ+ * Medium	4.19	1.70	**0.014**	[0.87; 7.51]
Sexual Orientation * Problems associated with Academic Performance	LGBTQ+ * One or more problems	−0.80	1.12	0.475	[−3.01; 1.40]
Sexual Orientation * Problems associated with Daily Organization	LGBTQ+ * One or more problems	−0.73	1.27	0.564	[−3.22; 1.76]
Sexual Orientation * Problems in Academic Community	LGBTQ+ * One or more problems	0.42	1.05	0.689	[−1.65; 2.49]
Sexual Orientation * Family and Affective Problems	LGBTQ+ * One or more problems	−0.85	0.93	0.356	[−2.67; 0.96]
Sexual Orientation * Financial Problems	LGBTQ+ * One or more problems	−0.59	0.93	0.527	[−2.41; 1.23]
Sexual Orientation * Social Support	LGBTQ+ * Not at all	−2.69	3.51	0.444	[−9.58; 4.20]
	LGBTQ+ * Little	−0.04	1.53	0.979	[−3.04; 2.96]
	LGBTQ+ * Medium	0.57	1.01	0.570	[−1.41; 2.55]
Sexual Orientation * Burnout	LGBTQ+ * With burnout	2.34	1.10	**0.034**	[0.18; 4.49]

Dependent variable: BDI. The reference categories selected are heterosexual (Sexual Orientation), male (Sex), no problems (for each problem variable), high satisfaction (for academic rankings satisfaction), high support (for Social Support), and no burnout (for Burnout). Statistically significant values are in bold. BDI: Beck’s Depression Inventory; B: Unstandardized regression coefficients; SD: Standard deviation; *t*: *t* test; *p*: *p*-value; CI: Confidence Intervals; *: Interaction between predictors.

**Table A14:** Parameter estimates from anxiety’s general linear model analysis.

Predictor	Parameter	B	SD	*p*	CI
Sexual Orientation	LGBTQ+	4.68	2.61	0.073	[−0.44; 9.79]
Sex	Other	7.54	5.09	0.139	[−2.45; 17.5]
	Female	3.16	0.73	**<0.001**	[1.73; 4.58]
Substance Abuse	One or more problems	4.55	1.32	**0.001**	[1.96; 7.15]
Satisfaction with Academic Rankings	Not at all	4.11	1.58	**0.009**	[1.01; 7.22]
	Little	2.43	1.06	**0.022**	[0.35; 4.51]
	Medium	1.57	0.96	0.100	[−0.30; 3.45]
Problems associated with Academic Performance	One or more problems	3.38	0.59	**<0.001**	[2.23; 4.54]
Problems associated with Daily Organization	One or more problems	3.48	0.61	**<0.001**	[2.28; 4.68]
Problems in Academic Community	One or more problems	2.48	0.62	**<0.001**	[1.26; 3.70]
Family and Affective Problems	One or more problems	2.80	0.51	**<0.001**	[1.81; 3.80]
Financial Problems	One or more problems	0.87	0.53	0.100	[−0.17; 1.91]
Social Support	Not at all	14.5	2.68	**<0.001**	[9.26; 19.8]
	Little	9.87	0.93	**<0.001**	[8.05; 11.7]
	Medium	5.20	0.55	**<0.001**	[4.13; 6.28]
Burnout	With burnout	9.48	0.66	**<0.001**	[8.19; 10.8]
Sexual Orientation * Sex	LGBTQ+ * Other	−2.81	5.93	0.636	[−14.4; 8.82]
	LGBTQ+ * Female	−2.65	1.48	0.073	[−5.55; 0.25]
Sexual Orientation * Substance Abuse	LGBTQ+ * One or more problems	−5.03	2.35	**0.032**	[−9.64; 0.43]
Sexual Orientation * Satisfaction with Academic Rankings	LGBTQ+ *Not at all	6.78	3.50	0.053	[−0.10; 13.6]
	LGBTQ+ * Little	3.58	2.64	0.175	[−1.59; 8.76]
	LGBTQ+ * Medium	2.10	2.33	0.368	[−2.47; 6.67]
Sexual Orientation * Problems associated with Academic Performance	LGBTQ+ * One or more problems	−1.82	1.55	0.239	[−4.85; 1.21]
Sexual Orientation * Problems associated with Daily Organization	LGBTQ+ * One or more problems	−2.91	1.75	0.096	[−6.33; 0.52]
Sexual Orientation * Problems in Academic Community	LGBTQ+ * One or more problems	−2.22	1.45	0.126	[−5.07; 0.62]
Sexual Orientation * Family and Affective Problems	LGBTQ+ * One or more problems	−2.33	1.27	0.068	[−4.83; 0.17]
Sexual Orientation * Financial Problems	LGBTQ+ * One or more problems	0.31	1.28	0.808	[−2.19;2.82]
Sexual Orientation * Social Support	LGBTQ+ * Not at all	−4.76	4.83	0.325	[−14.2; 4.72]
	LGBTQ+ * Little	−0.56	2.10	0.791	[−4.68; 3.57]
	LGBTQ+ * Medium	−0.30	1.39	0.829	[−3.02; 2.42]
Sexual Orientation * Burnout	LGBTQ+ * With burnout	1.53	1.51	0.311	[−1.43; 4.49]

Dependent variable: STAI Y-1. The reference categories selected are heterosexual (Sexual Orientation), male (Sex), no problems (for each problem variable), high satisfaction (for academic rankings satisfaction), high support (for Social Support), and no burnout (for Burnout). Statistically significant values are in bold. STAI: State-Trait Anxiety Inventory; B: Unstandardized regression coefficients; SD: Standard deviation; *t*: *t* test; *p*: *p*-value; CI: Confidence Intervals; *: Interaction between predictors.
